# [(*Z*)-*O*-Methyl *N*-(3-chloro­phen­yl)thio­carbamato-κ*S*](tricyclo­hexyl­phosphine-κ*P*)gold(I)

**DOI:** 10.1107/S1600536810010445

**Published:** 2010-03-27

**Authors:** Primjira P. Tadbuppa, Edward R. T. Tiekink

**Affiliations:** a Department of Chemistry, National University of Singapore, Singapore 117543; b Department of Chemistry, University of Malaya, 50603 Kuala Lumpur, Malaysia

## Abstract

Two independent mol­ecules comprise the asymmetric unit of the title compound, [Au(C_8_H_7_ClNOS)(C_18_H_33_P)], which differ in the relative orientations of each of the cyclo­hexyl groups as well as the benzene ring. In each mol­ecule, the Au atom is coordinated within a *S*,*P*-donor set that defines a slightly distorted linear geometry [S—Au—P = 175.10 (5) and 177.26 (5)° for the two mol­ecules], with the distortion due in part to the close intra­molecular approach of the O atom [Au⋯O contacts = 3.054 (4) and 3.013 (4) Å, respectively, for the two mol­ecules].

## Related literature

For the structural systematics and luminescence properties of phosphinegold(I) carbonimidothio­ates, see: Ho *et al.* (2006 ▶); Ho & Tiekink (2007 ▶); Kuan *et al.* (2008 ▶). For the synthesis, see: Hall *et al.* (1993 ▶).
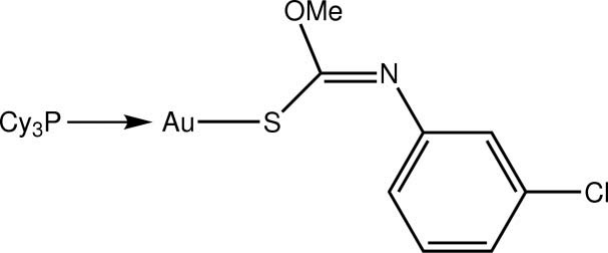


## Experimental

### 

#### Crystal data

[Au(C_8_H_7_ClNOS)(C_18_H_33_P)]
*M* *_r_* = 678.04
Monoclinic, 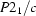

*a* = 19.1964 (8) Å
*b* = 11.7855 (5) Å
*c* = 26.0594 (11) Å
β = 107.328 (1)°
*V* = 5628.1 (4) Å^3^
*Z* = 8
Mo *K*α radiation
μ = 5.47 mm^−1^
*T* = 223 K
0.32 × 0.09 × 0.08 mm

#### Data collection

Bruker SMART CCD diffractometer
Absorption correction: multi-scan (*SADABS*; Bruker, 2000 ▶) *T*_min_ = 0.120, *T*_max_ = 1.000
39394 measured reflections
12920 independent reflections
9743 reflections with *I* > 2σ(*I*)
*R*_int_ = 0.052

#### Refinement

*R*[*F* ^2^ > 2σ(*F* ^2^)] = 0.036
*wR*(*F* ^2^) = 0.103
*S* = 1.05
12920 reflections
579 parameters
H-atom parameters constrained
Δρ_max_ = 0.90 e Å^−3^
Δρ_min_ = −1.26 e Å^−3^

### 

Data collection: *SMART* (Bruker, 2000 ▶); cell refinement: *SAINT* (Bruker, 2000 ▶); data reduction: *SAINT*; program(s) used to solve structure: *PATTY* in *DIRDIF92* (Beurskens *et al.*, 1992 ▶); program(s) used to refine structure: *SHELXL97* (Sheldrick, 2008 ▶); molecular graphics: *ORTEP-3* (Farrugia, 1997 ▶), *DIAMOND* (Brandenburg, 2006 ▶) and *Qmol* (Gans & Shalloway, 2001 ▶); software used to prepare material for publication: *publCIF* (Westrip, 2010 ▶).

## Supplementary Material

Crystal structure: contains datablocks global, I. DOI: 10.1107/S1600536810010445/pv2267sup1.cif

Structure factors: contains datablocks I. DOI: 10.1107/S1600536810010445/pv2267Isup2.hkl

Additional supplementary materials:  crystallographic information; 3D view; checkCIF report

## Figures and Tables

**Table 1:** Selected bond lengths (Å)

Au1—P1	2.2565 (14)
Au1—S1	2.2982 (14)
S1—C1	1.741 (5)
N1—C1	1.257 (6)
Au2—P2	2.2572 (14)
Au2—S2	2.2949 (15)
S2—C27	1.748 (5)
N2—C27	1.255 (6)

**Fig. 1.**
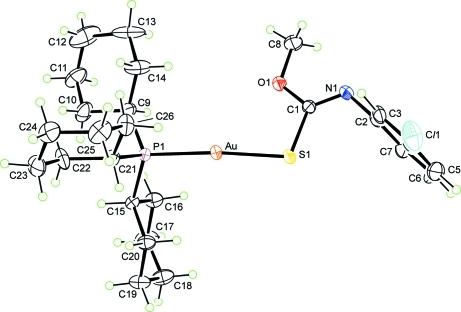
Molecular structure of the first independent molecule comprising the asymmetric unit in (I) showing atom-labelling scheme (the C4 atom is obscured by Cl1) and displacement ellipsoids at the 35% probability level.

**Fig. 2.**
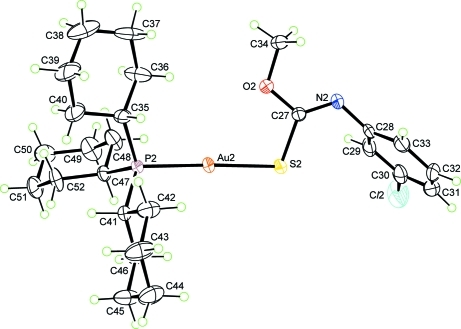
Molecular structure of the second independent molecule comprising the asymmetric unit in (I) showing atom-labelling scheme and displacement ellipsoids at the 35% probability level.

**Fig. 3.**
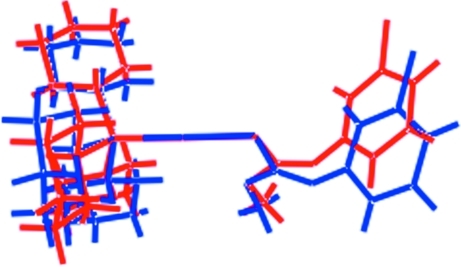
Overlay diagram showing the different conformations for molecule a (red image) and molecule b (blue image).

## supplementary crystallographic information

### Comment

Molecules of the type R_3_PAu[SC(OR')═NR''], for R, R' and R'' = alkyl and aryl, attract interest in terms of crystal engineering and luminescence studies (Ho *et al.* 2006; Ho & Tiekink, 2007; Kuan *et al.*, 2008). It was in this context that the synthesis and characterisation of the title compound, (I), was investigated.

Two independent molecules, *a* (Fig. 1) and *b* (Fig. 2), comprise the asymmetric unit of (I). As seen from the overlay diagram, Fig. 3 (Gans & Shalloway, 2001), the molecules differ in terms of the relative orientations of the cyclohexyl and benzene rings. Each of the gold atoms exist within a *SP* donor set, Table 1, with small deviations from the ideal linearity ascribed to the close approach of the O atom [Au···O contacts = 3.054 (4) and 3.013 (4) Å for molecules *a* and *b*, respectively]. The ligand is coordinating as a thiolate as evidenced by the C–S and C═N bond distances, Table 1. No specific intermolecular interactions are noted in the crystal packing.

### Experimental

Compound (I) was prepared following the standard literature procedure from the reaction of Cy_3_PAuCl and MeOC(═S)N(H)(C_6_H_4_Cl-3) in the presence of NaOH (Hall *et al.*, 1993). Crystals were obtained by the slow evaporation of a CH_2_Cl_2_/hexane (3/1) solution held at room temperature.

### Refinement

The H atoms were geometrically placed (C—H = 0.94-0.99 Å) and refined as riding with *U*_iso_(H) = 1.2-1.5*U*_eq_(C). While high thermal motion is noted for some of the cyclohexyl rings, multiple positions for these could not be resolved in the refinement. The maximum and minimum residual electron density peaks of 0.90 and 1.26 e Å^-3^, respectively, were located 0.38 Å and 0.87 Å from the C5 and Au1 atoms, respectively.

### Crystal data

**Table:**

[Au(C_8_H_7_ClNOS)(C_18_H_33_P)]	*F*(000) = 2704
*M**_r_* = 678.04	*D*_x_ = 1.600 Mg m^−^^3^
Monoclinic, *P*2_1_/*c*	Mo *K*α radiation, λ = 0.71069 Å
Hall symbol: -P 2ybc	Cell parameters from 7254 reflections
*a* = 19.1964 (8) Å	θ = 2.2–27.8°
*b* = 11.7855 (5) Å	µ = 5.47 mm^−^^1^
*c* = 26.0594 (11) Å	*T* = 223 K
β = 107.328 (1)°	Block, colourless
*V* = 5628.1 (4) Å^3^	0.32 × 0.09 × 0.08 mm
*Z* = 8	

### Data collection

**Table:**

Bruker SMART CCD diffractometer	12920 independent reflections
Radiation source: fine-focus sealed tube	9743 reflections with *I* > 2σ(*I*)
graphite	*R*_int_ = 0.052
ω scans	θ_max_ = 27.5°, θ_min_ = 1.1°
Absorption correction: multi-scan (*SADABS*; Bruker, 2000)	*h* = −24→23
*T*_min_ = 0.120, *T*_max_ = 1.000	*k* = −15→15
39394 measured reflections	*l* = −33→23

### Refinement

**Table:**

Refinement on *F*^2^	Primary atom site location: structure-invariant direct methods
Least-squares matrix: full	Secondary atom site location: difference Fourier map
*R*[*F*^2^ > 2σ(*F*^2^)] = 0.036	Hydrogen site location: inferred from neighbouring sites
*wR*(*F*^2^) = 0.103	H-atom parameters constrained
*S* = 1.05	*w* = 1/[σ^2^(*F*_o_^2^) + (0.0443*P*)^2^ + 1.5842*P*] where *P* = (*F*_o_^2^ + 2*F*_c_^2^)/3
12920 reflections	(Δ/σ)_max_ = 0.002
579 parameters	Δρ_max_ = 0.90 e Å^−^^3^
0 restraints	Δρ_min_ = −1.26 e Å^−^^3^

### Special details

**Table:**

Geometry. All s.u.'s (except the s.u. in the dihedral angle between two l.s. planes) are estimated using the full covariance matrix. The cell s.u.'s are taken into account individually in the estimation of s.u.'s in distances, angles and torsion angles; correlations between s.u.'s in cell parameters are only used when they are defined by crystal symmetry. An approximate (isotropic) treatment of cell s.u.'s is used for estimating s.u.'s involving l.s. planes.
Refinement. Refinement of *F*^2^ against ALL reflections. The weighted *R*-factor wR and goodness of fit *S* are based on *F*^2^, conventional *R*-factors *R* are based on *F*, with *F* set to zero for negative *F*^2^. The threshold expression of *F*^2^ > 2σ(*F*^2^) is used only for calculating *R*-factors(gt) etc. and is not relevant to the choice of reflections for refinement. *R*-factors based on *F*^2^ are statistically about twice as large as those based on *F*, and *R*- factors based on ALL data will be even larger.

### Fractional atomic coordinates and isotropic or equivalent isotropic displacement parameters (Å^2^)

**Table:**

	*x*	*y*	*z*	*U*_iso_*/*U*_eq_	
Au1	0.033556 (11)	0.169486 (16)	0.215414 (8)	0.02957 (7)	
Cl1	−0.33553 (9)	0.21770 (19)	0.02053 (7)	0.0666 (5)	
S1	−0.03791 (8)	0.22019 (11)	0.13099 (6)	0.0395 (4)	
P1	0.10367 (7)	0.13548 (10)	0.30030 (6)	0.0260 (3)	
O1	0.0192 (2)	0.0287 (3)	0.11442 (15)	0.0363 (9)	
N1	−0.0744 (2)	0.0891 (4)	0.04332 (18)	0.0335 (10)	
C1	−0.0336 (3)	0.1047 (4)	0.0904 (2)	0.0276 (11)	
C2	−0.1303 (3)	0.1714 (4)	0.0221 (2)	0.0320 (12)	
C3	−0.1985 (3)	0.1575 (5)	0.0297 (2)	0.0362 (13)	
H3	−0.2079	0.0943	0.0486	0.043*	
C4	−0.2516 (3)	0.2367 (6)	0.0095 (2)	0.0418 (15)	
C5	−0.2408 (3)	0.3277 (5)	−0.0194 (3)	0.0474 (16)	
H5	−0.2782	0.3811	−0.0328	0.057*	
C6	−0.1747 (4)	0.3401 (5)	−0.0285 (3)	0.0535 (18)	
H6	−0.1671	0.4019	−0.0489	0.064*	
C7	−0.1187 (3)	0.2630 (5)	−0.0081 (2)	0.0429 (15)	
H7	−0.0735	0.2725	−0.0146	0.051*	
C8	0.0245 (4)	−0.0697 (5)	0.0832 (3)	0.0500 (17)	
H8A	−0.0213	−0.1109	0.0739	0.075*	
H8B	0.0634	−0.1185	0.1040	0.075*	
H8C	0.0350	−0.0462	0.0506	0.075*	
C9	0.1636 (3)	0.0117 (4)	0.3036 (2)	0.0373 (13)	
H9	0.1903	0.0286	0.2774	0.045*	
C10	0.2232 (4)	−0.0090 (6)	0.3564 (3)	0.0561 (18)	
H10A	0.2521	0.0604	0.3668	0.067*	
H10B	0.2004	−0.0258	0.3846	0.067*	
C11	0.2734 (4)	−0.1055 (5)	0.3531 (3)	0.063 (2)	
H11A	0.3021	−0.0843	0.3291	0.076*	
H11B	0.3073	−0.1202	0.3888	0.076*	
C12	0.2318 (4)	−0.2082 (6)	0.3330 (4)	0.093 (3)	
H12A	0.2088	−0.2345	0.3598	0.112*	
H12B	0.2656	−0.2674	0.3290	0.112*	
C13	0.1731 (4)	−0.1935 (5)	0.2798 (4)	0.083 (3)	
H13A	0.1450	−0.2639	0.2705	0.100*	
H13B	0.1959	−0.1780	0.2515	0.100*	
C14	0.1214 (4)	−0.0946 (5)	0.2828 (3)	0.066 (2)	
H14A	0.0878	−0.0802	0.2469	0.079*	
H14B	0.0924	−0.1159	0.3065	0.079*	
C15	0.1663 (3)	0.2562 (4)	0.3241 (2)	0.0273 (11)	
H15	0.1951	0.2424	0.3619	0.033*	
C16	0.2182 (3)	0.2680 (4)	0.2906 (3)	0.0393 (14)	
H16A	0.1901	0.2737	0.2525	0.047*	
H16B	0.2489	0.2000	0.2951	0.047*	
C17	0.2670 (4)	0.3731 (5)	0.3069 (3)	0.0567 (19)	
H17A	0.2977	0.3653	0.3442	0.068*	
H17B	0.2991	0.3795	0.2840	0.068*	
C18	0.2202 (4)	0.4797 (5)	0.3012 (3)	0.0548 (18)	
H18A	0.2516	0.5466	0.3115	0.066*	
H18B	0.1903	0.4889	0.2637	0.066*	
C19	0.1720 (4)	0.4693 (5)	0.3367 (3)	0.0524 (17)	
H19A	0.1422	0.5379	0.3335	0.063*	
H19B	0.2022	0.4626	0.3742	0.063*	
C20	0.1219 (3)	0.3651 (4)	0.3214 (3)	0.0383 (14)	
H20A	0.0921	0.3588	0.3459	0.046*	
H20B	0.0889	0.3748	0.2849	0.046*	
C21	0.0492 (3)	0.1226 (5)	0.3471 (2)	0.0345 (13)	
H21	0.0283	0.1991	0.3480	0.041*	
C22	0.0919 (3)	0.0974 (6)	0.4055 (2)	0.0460 (15)	
H22A	0.1304	0.1544	0.4182	0.055*	
H22B	0.1152	0.0229	0.4076	0.055*	
C23	0.0422 (4)	0.0986 (6)	0.4416 (3)	0.0542 (18)	
H23A	0.0703	0.0753	0.4780	0.065*	
H23B	0.0247	0.1761	0.4436	0.065*	
C24	−0.0217 (4)	0.0211 (5)	0.4212 (3)	0.0526 (17)	
H24A	−0.0538	0.0283	0.4441	0.063*	
H24B	−0.0044	−0.0575	0.4234	0.063*	
C25	−0.0645 (3)	0.0472 (6)	0.3641 (3)	0.0573 (19)	
H25A	−0.0869	0.1224	0.3625	0.069*	
H25B	−0.1037	−0.0086	0.3516	0.069*	
C26	−0.0163 (3)	0.0450 (5)	0.3270 (2)	0.0463 (15)	
H26A	0.0005	−0.0328	0.3246	0.056*	
H26B	−0.0451	0.0688	0.2909	0.056*	
Au2	0.558501 (11)	0.649071 (17)	0.747201 (9)	0.03400 (7)	
Cl2	0.71112 (14)	0.8119 (2)	1.02912 (9)	0.0950 (8)	
S2	0.64142 (9)	0.69232 (12)	0.82842 (6)	0.0457 (4)	
P2	0.47804 (7)	0.61468 (11)	0.66583 (6)	0.0289 (3)	
O2	0.67747 (19)	0.4891 (3)	0.80699 (15)	0.0380 (9)	
N2	0.7584 (2)	0.5672 (4)	0.88067 (19)	0.0383 (12)	
C27	0.7002 (3)	0.5755 (4)	0.8428 (2)	0.0319 (12)	
C28	0.7795 (3)	0.6585 (4)	0.9169 (3)	0.0391 (15)	
C29	0.7394 (3)	0.6862 (5)	0.9526 (2)	0.0459 (15)	
H29	0.6974	0.6449	0.9522	0.055*	
C30	0.7625 (4)	0.7735 (6)	0.9876 (3)	0.0558 (18)	
C31	0.8228 (5)	0.8372 (5)	0.9890 (3)	0.065 (2)	
H31	0.8364	0.8993	1.0125	0.078*	
C32	0.8629 (4)	0.8083 (5)	0.9551 (3)	0.065 (2)	
H32	0.9054	0.8494	0.9565	0.078*	
C33	0.8417 (3)	0.7192 (5)	0.9189 (3)	0.0498 (17)	
H33	0.8695	0.7003	0.8959	0.060*	
C34	0.7241 (3)	0.3915 (4)	0.8155 (3)	0.0430 (15)	
H34A	0.7720	0.4136	0.8137	0.065*	
H34B	0.7033	0.3353	0.7880	0.065*	
H34C	0.7285	0.3595	0.8506	0.065*	
C35	0.5094 (3)	0.4928 (4)	0.6342 (2)	0.0399 (14)	
H35	0.5564	0.5188	0.6298	0.048*	
C36	0.5299 (5)	0.3900 (5)	0.6697 (3)	0.069 (2)	
H36A	0.4862	0.3588	0.6765	0.083*	
H36B	0.5642	0.4121	0.7043	0.083*	
C37	0.5648 (5)	0.2992 (6)	0.6436 (4)	0.083 (3)	
H37A	0.6134	0.3252	0.6438	0.100*	
H37B	0.5712	0.2300	0.6653	0.100*	
C38	0.5219 (5)	0.2710 (6)	0.5873 (4)	0.087 (3)	
H38A	0.5509	0.2207	0.5717	0.105*	
H38B	0.4777	0.2299	0.5876	0.105*	
C39	0.5015 (5)	0.3717 (6)	0.5537 (4)	0.078 (3)	
H39A	0.4685	0.3494	0.5186	0.094*	
H39B	0.5454	0.4044	0.5477	0.094*	
C40	0.4645 (4)	0.4612 (6)	0.5781 (3)	0.0598 (19)	
H40A	0.4562	0.5291	0.5554	0.072*	
H40B	0.4169	0.4328	0.5790	0.072*	
C41	0.4746 (3)	0.7341 (4)	0.6197 (2)	0.0295 (12)	
H41	0.4376	0.7160	0.5851	0.035*	
C42	0.5479 (3)	0.7523 (5)	0.6085 (3)	0.0514 (18)	
H42A	0.5861	0.7636	0.6427	0.062*	
H42B	0.5602	0.6840	0.5916	0.062*	
C43	0.5467 (4)	0.8532 (5)	0.5724 (4)	0.068 (2)	
H43A	0.5956	0.8644	0.5688	0.082*	
H43B	0.5133	0.8373	0.5366	0.082*	
C44	0.5228 (4)	0.9609 (5)	0.5937 (3)	0.067 (2)	
H44A	0.5593	0.9827	0.6273	0.080*	
H44B	0.5191	1.0223	0.5676	0.080*	
C45	0.4507 (4)	0.9440 (5)	0.6034 (3)	0.063 (2)	
H45A	0.4132	0.9311	0.5690	0.076*	
H45B	0.4376	1.0130	0.6194	0.076*	
C46	0.4518 (3)	0.8432 (4)	0.6408 (3)	0.0468 (16)	
H46A	0.4857	0.8596	0.6764	0.056*	
H46B	0.4030	0.8334	0.6449	0.056*	
C47	0.3847 (3)	0.5955 (4)	0.6704 (2)	0.0326 (12)	
H47	0.3735	0.6664	0.6867	0.039*	
C48	0.3817 (3)	0.5027 (6)	0.7098 (3)	0.0563 (18)	
H48A	0.4188	0.5171	0.7442	0.068*	
H48B	0.3928	0.4296	0.6961	0.068*	
C49	0.3071 (3)	0.4967 (7)	0.7186 (3)	0.064 (2)	
H49A	0.3060	0.4326	0.7423	0.077*	
H49B	0.2987	0.5663	0.7365	0.077*	
C50	0.2468 (3)	0.4829 (5)	0.6664 (3)	0.0575 (19)	
H50A	0.2514	0.4089	0.6506	0.069*	
H50B	0.1994	0.4855	0.6734	0.069*	
C51	0.2505 (3)	0.5755 (6)	0.6276 (3)	0.0562 (18)	
H51A	0.2394	0.6483	0.6417	0.067*	
H51B	0.2132	0.5617	0.5933	0.067*	
C52	0.3248 (3)	0.5836 (6)	0.6180 (2)	0.0507 (17)	
H52A	0.3333	0.5153	0.5992	0.061*	
H52B	0.3255	0.6493	0.5951	0.061*	

### Atomic displacement parameters (Å^2^)

**Table:**

	*U*^11^	*U*^22^	*U*^33^	*U*^12^	*U*^13^	*U*^23^
Au1	0.03475 (13)	0.03001 (11)	0.02073 (11)	−0.00016 (8)	0.00333 (9)	−0.00099 (8)
Cl1	0.0336 (9)	0.1165 (16)	0.0482 (11)	0.0002 (9)	0.0097 (8)	−0.0057 (10)
S1	0.0525 (9)	0.0324 (7)	0.0236 (8)	0.0111 (6)	−0.0039 (7)	−0.0048 (6)
P1	0.0300 (7)	0.0245 (6)	0.0224 (7)	0.0008 (5)	0.0061 (6)	0.0017 (5)
O1	0.041 (2)	0.0328 (19)	0.030 (2)	0.0068 (16)	0.0014 (17)	−0.0036 (16)
N1	0.040 (3)	0.032 (2)	0.026 (3)	0.0017 (19)	0.007 (2)	−0.0068 (19)
C1	0.032 (3)	0.027 (3)	0.025 (3)	0.003 (2)	0.010 (2)	0.000 (2)
C2	0.034 (3)	0.036 (3)	0.021 (3)	0.001 (2)	−0.001 (2)	−0.006 (2)
C3	0.041 (3)	0.045 (3)	0.020 (3)	−0.008 (3)	0.006 (2)	−0.001 (2)
C4	0.029 (3)	0.065 (4)	0.025 (3)	0.003 (3)	−0.001 (3)	−0.009 (3)
C5	0.036 (4)	0.046 (4)	0.050 (4)	0.008 (3)	−0.002 (3)	0.001 (3)
C6	0.056 (4)	0.037 (3)	0.058 (5)	0.000 (3)	0.003 (4)	0.021 (3)
C7	0.036 (3)	0.049 (3)	0.041 (4)	−0.005 (3)	0.008 (3)	0.007 (3)
C8	0.056 (4)	0.037 (3)	0.053 (4)	0.015 (3)	0.011 (3)	−0.004 (3)
C9	0.044 (3)	0.029 (3)	0.040 (4)	0.004 (2)	0.015 (3)	0.002 (2)
C10	0.061 (4)	0.054 (4)	0.053 (4)	0.027 (3)	0.018 (4)	0.015 (3)
C11	0.066 (5)	0.049 (4)	0.083 (6)	0.025 (4)	0.033 (4)	0.020 (4)
C12	0.051 (5)	0.056 (5)	0.172 (10)	0.023 (4)	0.032 (6)	0.030 (6)
C13	0.083 (6)	0.026 (3)	0.162 (10)	−0.014 (3)	0.069 (7)	−0.028 (5)
C14	0.054 (4)	0.031 (3)	0.111 (7)	−0.008 (3)	0.023 (4)	−0.009 (4)
C15	0.027 (3)	0.025 (2)	0.030 (3)	0.002 (2)	0.009 (2)	0.003 (2)
C16	0.038 (3)	0.035 (3)	0.051 (4)	0.001 (2)	0.022 (3)	−0.001 (3)
C17	0.048 (4)	0.045 (4)	0.088 (6)	−0.011 (3)	0.035 (4)	−0.002 (4)
C18	0.056 (4)	0.033 (3)	0.082 (5)	−0.006 (3)	0.030 (4)	0.004 (3)
C19	0.063 (4)	0.031 (3)	0.065 (5)	−0.003 (3)	0.021 (4)	−0.010 (3)
C20	0.040 (3)	0.031 (3)	0.048 (4)	0.002 (2)	0.020 (3)	−0.003 (3)
C21	0.035 (3)	0.037 (3)	0.032 (3)	−0.003 (2)	0.010 (3)	0.005 (2)
C22	0.047 (4)	0.067 (4)	0.023 (3)	−0.013 (3)	0.008 (3)	−0.005 (3)
C23	0.073 (5)	0.062 (4)	0.039 (4)	−0.012 (4)	0.032 (4)	0.000 (3)
C24	0.059 (4)	0.054 (4)	0.060 (5)	0.002 (3)	0.042 (4)	0.007 (3)
C25	0.040 (4)	0.064 (4)	0.077 (5)	−0.001 (3)	0.031 (4)	0.016 (4)
C26	0.034 (3)	0.063 (4)	0.040 (4)	−0.007 (3)	0.009 (3)	0.007 (3)
Au2	0.02620 (12)	0.03448 (12)	0.03323 (14)	0.00288 (8)	−0.00351 (9)	−0.00166 (9)
Cl2	0.0986 (18)	0.131 (2)	0.0464 (13)	0.0114 (15)	0.0074 (12)	−0.0290 (13)
S2	0.0438 (9)	0.0354 (7)	0.0410 (9)	0.0104 (6)	−0.0135 (7)	−0.0075 (7)
P2	0.0234 (7)	0.0268 (6)	0.0313 (8)	0.0025 (5)	0.0002 (6)	−0.0014 (6)
O2	0.035 (2)	0.0267 (18)	0.043 (2)	0.0008 (15)	−0.0019 (18)	−0.0025 (17)
N2	0.031 (3)	0.033 (2)	0.039 (3)	0.0005 (19)	−0.006 (2)	0.002 (2)
C27	0.030 (3)	0.026 (3)	0.036 (3)	−0.002 (2)	0.004 (2)	0.004 (2)
C28	0.027 (3)	0.032 (3)	0.044 (4)	0.003 (2)	−0.012 (3)	0.008 (2)
C29	0.038 (4)	0.057 (4)	0.032 (4)	−0.001 (3)	−0.005 (3)	0.002 (3)
C30	0.053 (4)	0.061 (4)	0.037 (4)	0.009 (3)	−0.012 (3)	−0.004 (3)
C31	0.072 (6)	0.041 (4)	0.056 (5)	0.007 (3)	−0.020 (4)	−0.005 (3)
C32	0.043 (4)	0.040 (4)	0.085 (6)	−0.015 (3)	−0.021 (4)	0.009 (4)
C33	0.036 (4)	0.043 (3)	0.061 (5)	−0.001 (3)	0.000 (3)	0.012 (3)
C34	0.041 (3)	0.027 (3)	0.058 (4)	0.003 (2)	0.010 (3)	−0.002 (3)
C35	0.036 (3)	0.029 (3)	0.053 (4)	0.008 (2)	0.012 (3)	−0.004 (3)
C36	0.091 (6)	0.042 (4)	0.080 (6)	0.027 (4)	0.033 (5)	0.016 (4)
C37	0.096 (7)	0.033 (4)	0.140 (9)	0.033 (4)	0.064 (7)	0.021 (5)
C38	0.112 (7)	0.042 (4)	0.135 (9)	−0.002 (4)	0.079 (7)	−0.027 (5)
C39	0.094 (6)	0.053 (4)	0.092 (7)	0.013 (4)	0.032 (5)	−0.035 (4)
C40	0.060 (4)	0.052 (4)	0.065 (5)	0.008 (3)	0.014 (4)	−0.026 (4)
C41	0.026 (3)	0.030 (3)	0.029 (3)	0.000 (2)	0.003 (2)	−0.001 (2)
C42	0.041 (4)	0.038 (3)	0.084 (5)	0.001 (3)	0.033 (4)	0.000 (3)
C43	0.082 (6)	0.039 (4)	0.104 (7)	−0.010 (3)	0.060 (5)	0.000 (4)
C44	0.069 (5)	0.038 (4)	0.100 (7)	−0.014 (3)	0.037 (5)	−0.003 (4)
C45	0.067 (5)	0.032 (3)	0.099 (6)	0.011 (3)	0.038 (5)	0.010 (4)
C46	0.040 (4)	0.030 (3)	0.072 (5)	0.004 (2)	0.019 (3)	0.001 (3)
C47	0.021 (3)	0.036 (3)	0.038 (3)	−0.001 (2)	0.004 (2)	0.001 (2)
C48	0.038 (4)	0.067 (4)	0.057 (5)	−0.002 (3)	0.004 (3)	0.025 (4)
C49	0.049 (4)	0.089 (5)	0.061 (5)	−0.005 (4)	0.026 (4)	0.021 (4)
C50	0.036 (4)	0.051 (4)	0.090 (6)	−0.004 (3)	0.026 (4)	0.007 (4)
C51	0.024 (3)	0.075 (5)	0.063 (5)	−0.005 (3)	0.003 (3)	0.004 (4)
C52	0.023 (3)	0.086 (5)	0.037 (4)	−0.002 (3)	0.000 (3)	0.011 (3)

### Geometric parameters (Å, °)

**Table:**

Au1—P1	2.2565 (14)	Au2—P2	2.2572 (14)
Au1—S1	2.2982 (14)	Au2—S2	2.2949 (15)
Cl1—C4	1.733 (6)	Cl2—C30	1.727 (8)
S1—C1	1.741 (5)	S2—C27	1.748 (5)
P1—C21	1.834 (6)	P2—C41	1.839 (5)
P1—C9	1.844 (5)	P2—C35	1.844 (5)
P1—C15	1.847 (5)	P2—C47	1.845 (5)
O1—C1	1.357 (6)	O2—C27	1.362 (6)
O1—C8	1.438 (6)	O2—C34	1.434 (6)
N1—C1	1.257 (6)	N2—C27	1.255 (6)
N1—C2	1.430 (7)	N2—C28	1.409 (7)
C2—C3	1.391 (8)	C28—C33	1.379 (8)
C2—C7	1.392 (7)	C28—C29	1.411 (9)
C3—C4	1.367 (8)	C29—C30	1.358 (9)
C3—H3	0.9400	C29—H29	0.9400
C4—C5	1.360 (9)	C30—C31	1.372 (10)
C5—C6	1.366 (9)	C31—C32	1.377 (11)
C5—H5	0.9400	C31—H31	0.9400
C6—C7	1.387 (8)	C32—C33	1.390 (9)
C6—H6	0.9400	C32—H32	0.9400
C7—H7	0.9400	C33—H33	0.9400
C8—H8A	0.9700	C34—H34A	0.9700
C8—H8B	0.9700	C34—H34B	0.9700
C8—H8C	0.9700	C34—H34C	0.9700
C9—C14	1.503 (8)	C35—C36	1.503 (8)
C9—C10	1.524 (8)	C35—C40	1.507 (8)
C9—H9	0.9900	C35—H35	0.9900
C10—C11	1.509 (8)	C36—C37	1.526 (10)
C10—H10A	0.9800	C36—H36A	0.9800
C10—H10B	0.9800	C36—H36B	0.9800
C11—C12	1.459 (10)	C37—C38	1.490 (12)
C11—H11A	0.9800	C37—H37A	0.9800
C11—H11B	0.9800	C37—H37B	0.9800
C12—C13	1.513 (12)	C38—C39	1.458 (11)
C12—H12A	0.9800	C38—H38A	0.9800
C12—H12B	0.9800	C38—H38B	0.9800
C13—C14	1.549 (9)	C39—C40	1.513 (8)
C13—H13A	0.9800	C39—H39A	0.9800
C13—H13B	0.9800	C39—H39B	0.9800
C14—H14A	0.9800	C40—H40A	0.9800
C14—H14B	0.9800	C40—H40B	0.9800
C15—C16	1.514 (7)	C41—C46	1.515 (7)
C15—C20	1.530 (7)	C41—C42	1.535 (7)
C15—H15	0.9900	C41—H41	0.9900
C16—C17	1.535 (8)	C42—C43	1.512 (9)
C16—H16A	0.9800	C42—H42A	0.9800
C16—H16B	0.9800	C42—H42B	0.9800
C17—C18	1.526 (8)	C43—C44	1.509 (9)
C17—H17A	0.9800	C43—H43A	0.9800
C17—H17B	0.9800	C43—H43B	0.9800
C18—C19	1.495 (9)	C44—C45	1.494 (9)
C18—H18A	0.9800	C44—H44A	0.9800
C18—H18B	0.9800	C44—H44B	0.9800
C19—C20	1.538 (7)	C45—C46	1.533 (8)
C19—H19A	0.9800	C45—H45A	0.9800
C19—H19B	0.9800	C45—H45B	0.9800
C20—H20A	0.9800	C46—H46A	0.9800
C20—H20B	0.9800	C46—H46B	0.9800
C21—C26	1.517 (7)	C47—C52	1.506 (7)
C21—C22	1.526 (8)	C47—C48	1.513 (8)
C21—H21	0.9900	C47—H47	0.9900
C22—C23	1.527 (8)	C48—C49	1.517 (9)
C22—H22A	0.9800	C48—H48A	0.9800
C22—H22B	0.9800	C48—H48B	0.9800
C23—C24	1.496 (9)	C49—C50	1.509 (9)
C23—H23A	0.9800	C49—H49A	0.9800
C23—H23B	0.9800	C49—H49B	0.9800
C24—C25	1.502 (9)	C50—C51	1.504 (9)
C24—H24A	0.9800	C50—H50A	0.9800
C24—H24B	0.9800	C50—H50B	0.9800
C25—C26	1.523 (8)	C51—C52	1.524 (8)
C25—H25A	0.9800	C51—H51A	0.9800
C25—H25B	0.9800	C51—H51B	0.9800
C26—H26A	0.9800	C52—H52A	0.9800
C26—H26B	0.9800	C52—H52B	0.9800
			
P1—Au1—S1	175.10 (5)	P2—Au2—S2	177.26 (5)
C1—S1—Au1	104.79 (18)	C27—S2—Au2	104.42 (19)
C21—P1—C9	112.0 (3)	C41—P2—C35	104.9 (3)
C21—P1—C15	106.5 (2)	C41—P2—C47	106.8 (2)
C9—P1—C15	105.0 (2)	C35—P2—C47	112.7 (3)
C21—P1—Au1	112.04 (19)	C41—P2—Au2	110.99 (17)
C9—P1—Au1	111.61 (19)	C35—P2—Au2	109.64 (19)
C15—P1—Au1	109.31 (17)	C47—P2—Au2	111.58 (19)
C1—O1—C8	116.1 (4)	C27—O2—C34	115.8 (4)
C1—N1—C2	117.1 (4)	C27—N2—C28	118.5 (5)
N1—C1—O1	121.1 (5)	N2—C27—O2	120.6 (5)
N1—C1—S1	125.3 (4)	N2—C27—S2	126.7 (4)
O1—C1—S1	113.6 (4)	O2—C27—S2	112.8 (4)
C3—C2—C7	119.2 (5)	C33—C28—N2	119.6 (6)
C3—C2—N1	119.6 (5)	C33—C28—C29	119.4 (6)
C7—C2—N1	121.1 (5)	N2—C28—C29	121.0 (5)
C4—C3—C2	119.4 (5)	C30—C29—C28	119.1 (6)
C4—C3—H3	120.3	C30—C29—H29	120.5
C2—C3—H3	120.3	C28—C29—H29	120.5
C5—C4—C3	122.1 (6)	C29—C30—C31	122.4 (7)
C5—C4—Cl1	119.7 (5)	C29—C30—Cl2	119.1 (6)
C3—C4—Cl1	118.1 (5)	C31—C30—Cl2	118.3 (6)
C4—C5—C6	118.9 (6)	C30—C31—C32	118.5 (7)
C4—C5—H5	120.6	C30—C31—H31	120.8
C6—C5—H5	120.6	C32—C31—H31	120.8
C5—C6—C7	121.2 (6)	C31—C32—C33	121.0 (7)
C5—C6—H6	119.4	C31—C32—H32	119.5
C7—C6—H6	119.4	C33—C32—H32	119.5
C6—C7—C2	119.2 (6)	C28—C33—C32	119.6 (7)
C6—C7—H7	120.4	C28—C33—H33	120.2
C2—C7—H7	120.4	C32—C33—H33	120.2
O1—C8—H8A	109.5	O2—C34—H34A	109.5
O1—C8—H8B	109.5	O2—C34—H34B	109.5
H8A—C8—H8B	109.5	H34A—C34—H34B	109.5
O1—C8—H8C	109.5	O2—C34—H34C	109.5
H8A—C8—H8C	109.5	H34A—C34—H34C	109.5
H8B—C8—H8C	109.5	H34B—C34—H34C	109.5
C14—C9—C10	111.8 (5)	C36—C35—C40	111.5 (5)
C14—C9—P1	112.4 (4)	C36—C35—P2	114.5 (5)
C10—C9—P1	117.3 (4)	C40—C35—P2	117.2 (4)
C14—C9—H9	104.6	C36—C35—H35	103.9
C10—C9—H9	104.6	C40—C35—H35	103.9
P1—C9—H9	104.6	P2—C35—H35	103.9
C11—C10—C9	112.9 (6)	C35—C36—C37	111.1 (6)
C11—C10—H10A	109.0	C35—C36—H36A	109.4
C9—C10—H10A	109.0	C37—C36—H36A	109.4
C11—C10—H10B	109.0	C35—C36—H36B	109.4
C9—C10—H10B	109.0	C37—C36—H36B	109.4
H10A—C10—H10B	107.8	H36A—C36—H36B	108.0
C12—C11—C10	110.8 (6)	C38—C37—C36	114.1 (7)
C12—C11—H11A	109.5	C38—C37—H37A	108.7
C10—C11—H11A	109.5	C36—C37—H37A	108.7
C12—C11—H11B	109.5	C38—C37—H37B	108.7
C10—C11—H11B	109.5	C36—C37—H37B	108.7
H11A—C11—H11B	108.1	H37A—C37—H37B	107.6
C11—C12—C13	114.4 (6)	C39—C38—C37	112.4 (6)
C11—C12—H12A	108.7	C39—C38—H38A	109.1
C13—C12—H12A	108.7	C37—C38—H38A	109.1
C11—C12—H12B	108.7	C39—C38—H38B	109.1
C13—C12—H12B	108.7	C37—C38—H38B	109.1
H12A—C12—H12B	107.6	H38A—C38—H38B	107.9
C12—C13—C14	110.7 (7)	C38—C39—C40	113.1 (7)
C12—C13—H13A	109.5	C38—C39—H39A	109.0
C14—C13—H13A	109.5	C40—C39—H39A	109.0
C12—C13—H13B	109.5	C38—C39—H39B	109.0
C14—C13—H13B	109.5	C40—C39—H39B	109.0
H13A—C13—H13B	108.1	H39A—C39—H39B	107.8
C9—C14—C13	111.2 (6)	C35—C40—C39	112.1 (6)
C9—C14—H14A	109.4	C35—C40—H40A	109.2
C13—C14—H14A	109.4	C39—C40—H40A	109.2
C9—C14—H14B	109.4	C35—C40—H40B	109.2
C13—C14—H14B	109.4	C39—C40—H40B	109.2
H14A—C14—H14B	108.0	H40A—C40—H40B	107.9
C16—C15—C20	110.5 (4)	C46—C41—C42	109.3 (4)
C16—C15—P1	110.6 (4)	C46—C41—P2	111.7 (4)
C20—C15—P1	109.4 (4)	C42—C41—P2	112.0 (4)
C16—C15—H15	108.8	C46—C41—H41	107.9
C20—C15—H15	108.8	C42—C41—H41	107.9
P1—C15—H15	108.8	P2—C41—H41	107.9
C15—C16—C17	111.5 (5)	C43—C42—C41	112.5 (5)
C15—C16—H16A	109.3	C43—C42—H42A	109.1
C17—C16—H16A	109.3	C41—C42—H42A	109.1
C15—C16—H16B	109.3	C43—C42—H42B	109.1
C17—C16—H16B	109.3	C41—C42—H42B	109.1
H16A—C16—H16B	108.0	H42A—C42—H42B	107.8
C18—C17—C16	110.2 (5)	C44—C43—C42	112.6 (6)
C18—C17—H17A	109.6	C44—C43—H43A	109.1
C16—C17—H17A	109.6	C42—C43—H43A	109.1
C18—C17—H17B	109.6	C44—C43—H43B	109.1
C16—C17—H17B	109.6	C42—C43—H43B	109.1
H17A—C17—H17B	108.1	H43A—C43—H43B	107.8
C19—C18—C17	109.1 (5)	C45—C44—C43	110.4 (5)
C19—C18—H18A	109.9	C45—C44—H44A	109.6
C17—C18—H18A	109.9	C43—C44—H44A	109.6
C19—C18—H18B	109.9	C45—C44—H44B	109.6
C17—C18—H18B	109.9	C43—C44—H44B	109.6
H18A—C18—H18B	108.3	H44A—C44—H44B	108.1
C18—C19—C20	111.1 (5)	C44—C45—C46	111.9 (6)
C18—C19—H19A	109.4	C44—C45—H45A	109.2
C20—C19—H19A	109.4	C46—C45—H45A	109.2
C18—C19—H19B	109.4	C44—C45—H45B	109.2
C20—C19—H19B	109.4	C46—C45—H45B	109.2
H19A—C19—H19B	108.0	H45A—C45—H45B	107.9
C15—C20—C19	111.1 (5)	C41—C46—C45	112.2 (6)
C15—C20—H20A	109.4	C41—C46—H46A	109.2
C19—C20—H20A	109.4	C45—C46—H46A	109.2
C15—C20—H20B	109.4	C41—C46—H46B	109.2
C19—C20—H20B	109.4	C45—C46—H46B	109.2
H20A—C20—H20B	108.0	H46A—C46—H46B	107.9
C26—C21—C22	111.8 (5)	C52—C47—C48	112.1 (5)
C26—C21—P1	113.5 (4)	C52—C47—P2	116.5 (4)
C22—C21—P1	115.8 (4)	C48—C47—P2	111.5 (4)
C26—C21—H21	104.8	C52—C47—H47	105.2
C22—C21—H21	104.8	C48—C47—H47	105.2
P1—C21—H21	104.8	P2—C47—H47	105.2
C21—C22—C23	111.3 (5)	C47—C48—C49	111.4 (5)
C21—C22—H22A	109.4	C47—C48—H48A	109.4
C23—C22—H22A	109.4	C49—C48—H48A	109.4
C21—C22—H22B	109.4	C47—C48—H48B	109.4
C23—C22—H22B	109.4	C49—C48—H48B	109.4
H22A—C22—H22B	108.0	H48A—C48—H48B	108.0
C24—C23—C22	111.9 (5)	C50—C49—C48	112.0 (6)
C24—C23—H23A	109.2	C50—C49—H49A	109.2
C22—C23—H23A	109.2	C48—C49—H49A	109.2
C24—C23—H23B	109.2	C50—C49—H49B	109.2
C22—C23—H23B	109.2	C48—C49—H49B	109.2
H23A—C23—H23B	107.9	H49A—C49—H49B	107.9
C23—C24—C25	112.1 (5)	C51—C50—C49	110.4 (5)
C23—C24—H24A	109.2	C51—C50—H50A	109.6
C25—C24—H24A	109.2	C49—C50—H50A	109.6
C23—C24—H24B	109.2	C51—C50—H50B	109.6
C25—C24—H24B	109.2	C49—C50—H50B	109.6
H24A—C24—H24B	107.9	H50A—C50—H50B	108.1
C24—C25—C26	111.7 (5)	C50—C51—C52	113.0 (5)
C24—C25—H25A	109.3	C50—C51—H51A	109.0
C26—C25—H25A	109.3	C52—C51—H51A	109.0
C24—C25—H25B	109.3	C50—C51—H51B	109.0
C26—C25—H25B	109.3	C52—C51—H51B	109.0
H25A—C25—H25B	107.9	H51A—C51—H51B	107.8
C21—C26—C25	111.6 (5)	C47—C52—C51	110.8 (5)
C21—C26—H26A	109.3	C47—C52—H52A	109.5
C25—C26—H26A	109.3	C51—C52—H52A	109.5
C21—C26—H26B	109.3	C47—C52—H52B	109.5
C25—C26—H26B	109.3	C51—C52—H52B	109.5
H26A—C26—H26B	108.0	H52A—C52—H52B	108.1
			
C2—N1—C1—O1	177.6 (5)	C28—N2—C27—O2	−179.7 (5)
C2—N1—C1—S1	−2.0 (7)	C28—N2—C27—S2	1.6 (8)
C8—O1—C1—N1	−0.8 (7)	C34—O2—C27—N2	−0.4 (8)
C8—O1—C1—S1	178.9 (4)	C34—O2—C27—S2	178.5 (4)
Au1—S1—C1—N1	167.3 (4)	Au2—S2—C27—N2	172.1 (5)
Au1—S1—C1—O1	−12.3 (4)	Au2—S2—C27—O2	−6.7 (4)
C1—N1—C2—C3	−89.4 (6)	C27—N2—C28—C33	−114.0 (6)
C1—N1—C2—C7	93.4 (6)	C27—N2—C28—C29	68.4 (7)
C7—C2—C3—C4	−3.0 (8)	C33—C28—C29—C30	1.1 (8)
N1—C2—C3—C4	179.8 (5)	N2—C28—C29—C30	178.7 (5)
C2—C3—C4—C5	1.9 (9)	C28—C29—C30—C31	1.1 (10)
C2—C3—C4—Cl1	−179.1 (4)	C28—C29—C30—Cl2	176.9 (4)
C3—C4—C5—C6	0.2 (10)	C29—C30—C31—C32	−2.8 (10)
Cl1—C4—C5—C6	−178.8 (5)	Cl2—C30—C31—C32	−178.6 (5)
C4—C5—C6—C7	−1.2 (10)	C30—C31—C32—C33	2.3 (10)
C5—C6—C7—C2	0.1 (10)	N2—C28—C33—C32	−179.2 (5)
C3—C2—C7—C6	2.0 (9)	C29—C28—C33—C32	−1.6 (9)
N1—C2—C7—C6	179.2 (5)	C31—C32—C33—C28	−0.1 (10)
C21—P1—C9—C14	67.9 (5)	C41—P2—C35—C36	169.1 (5)
C15—P1—C9—C14	−177.0 (5)	C47—P2—C35—C36	−75.0 (5)
Au1—P1—C9—C14	−58.7 (5)	Au2—P2—C35—C36	49.9 (5)
C21—P1—C9—C10	−63.9 (5)	C41—P2—C35—C40	−57.4 (5)
C15—P1—C9—C10	51.2 (5)	C47—P2—C35—C40	58.4 (6)
Au1—P1—C9—C10	169.6 (4)	Au2—P2—C35—C40	−176.7 (4)
C14—C9—C10—C11	53.5 (8)	C40—C35—C36—C37	51.6 (8)
P1—C9—C10—C11	−174.5 (5)	P2—C35—C36—C37	−172.3 (6)
C9—C10—C11—C12	−53.4 (9)	C35—C36—C37—C38	−50.7 (10)
C10—C11—C12—C13	54.6 (10)	C36—C37—C38—C39	50.8 (10)
C11—C12—C13—C14	−54.2 (9)	C37—C38—C39—C40	−51.7 (10)
C10—C9—C14—C13	−52.3 (8)	C36—C35—C40—C39	−53.6 (8)
P1—C9—C14—C13	173.3 (6)	P2—C35—C40—C39	171.7 (5)
C12—C13—C14—C9	52.0 (9)	C38—C39—C40—C35	53.8 (10)
C21—P1—C15—C16	175.3 (4)	C35—P2—C41—C46	−178.2 (4)
C9—P1—C15—C16	56.4 (4)	C47—P2—C41—C46	62.0 (4)
Au1—P1—C15—C16	−63.5 (4)	Au2—P2—C41—C46	−59.9 (4)
C21—P1—C15—C20	−62.7 (4)	C35—P2—C41—C42	−55.2 (5)
C9—P1—C15—C20	178.4 (4)	C47—P2—C41—C42	−175.0 (4)
Au1—P1—C15—C20	58.5 (4)	Au2—P2—C41—C42	63.1 (4)
C20—C15—C16—C17	54.5 (6)	C46—C41—C42—C43	−53.3 (7)
P1—C15—C16—C17	175.8 (4)	P2—C41—C42—C43	−177.6 (5)
C15—C16—C17—C18	−57.9 (7)	C41—C42—C43—C44	54.6 (9)
C16—C17—C18—C19	59.7 (8)	C42—C43—C44—C45	−54.7 (9)
C17—C18—C19—C20	−59.7 (7)	C43—C44—C45—C46	55.2 (9)
C16—C15—C20—C19	−53.6 (7)	C42—C41—C46—C45	54.0 (7)
P1—C15—C20—C19	−175.6 (4)	P2—C41—C46—C45	178.5 (5)
C18—C19—C20—C15	57.2 (7)	C44—C45—C46—C41	−56.6 (8)
C9—P1—C21—C26	−78.6 (5)	C41—P2—C47—C52	52.6 (5)
C15—P1—C21—C26	167.1 (4)	C35—P2—C47—C52	−62.1 (5)
Au1—P1—C21—C26	47.7 (4)	Au2—P2—C47—C52	174.1 (4)
C9—P1—C21—C22	52.7 (5)	C41—P2—C47—C48	−177.1 (4)
C15—P1—C21—C22	−61.6 (5)	C35—P2—C47—C48	68.3 (5)
Au1—P1—C21—C22	179.0 (4)	Au2—P2—C47—C48	−55.6 (5)
C26—C21—C22—C23	−52.8 (7)	C52—C47—C48—C49	−54.3 (8)
P1—C21—C22—C23	175.1 (4)	P2—C47—C48—C49	173.0 (5)
C21—C22—C23—C24	53.7 (7)	C47—C48—C49—C50	55.0 (8)
C22—C23—C24—C25	−55.1 (8)	C48—C49—C50—C51	−54.7 (8)
C23—C24—C25—C26	55.2 (8)	C49—C50—C51—C52	54.6 (8)
C22—C21—C26—C25	53.2 (7)	C48—C47—C52—C51	53.3 (7)
P1—C21—C26—C25	−173.6 (4)	P2—C47—C52—C51	−176.6 (4)
C24—C25—C26—C21	−54.1 (7)	C50—C51—C52—C47	−54.1 (8)
